# Inhibition of type I interferon signaling is a conserved function of gamma-herpesvirus-encoded microRNAs

**DOI:** 10.1128/jvi.01579-25

**Published:** 2025-12-31

**Authors:** Devin N. Fachko, Yan Chen, Nikita S. Ivanov, Bonnie Goff, Brian Pendergrass, Darby G. Oldenburg, Ryan D. Estep, Scott W. Wong, Rebecca L. Skalsky

**Affiliations:** 1 Vaccine and Gene Therapy Institute 828705, Beaverton, Oregon, USA; 2 Gundersen Medical Foundation 543836 , La Crosse, Wisconsin, USA; 3 Oregon National Primate Research Center 88960, Beaverton, Oregon, USA; University of Virginia, Charlottesville, Virginia, USA

**Keywords:** JAK/STAT pathway, type I interferon, innate immunity, microRNAs, herpesvirus

## Abstract

Herpesviruses encode multiple factors that disarm innate immune signaling to evade host anti-viral responses. Several viral microRNAs expressed by Epstein-Barr virus (EBV) and Kaposi’s sarcoma-associated herpesvirus (KSHV) disrupt the induction of type I interferons (IFN) and/or the subsequent signaling events activated through type I IFN receptors. Here, we aimed to determine whether non-human primate (NHP) γ-herpesviruses (rhesus lymphocryptovirus [rLCV], rhesus rhadinovirus [RRV], and Japanese macaque rhadinovirus [JMRV]), closely related to EBV and KSHV, use similar microRNA (miRNA)-mediated strategies to regulate IFN responses. Through functional screens, we identified multiple viral miRNAs that attenuated type I IFN-mediated activation of an IFN-stimulated response element reporter and diminished expression of interferon-stimulated genes (ISGs). Infection of primary cells with miRNA-deficient rLCVs resulted in augmented expression of ISGs. Abrogation of EBV BART miRNA homologs from rLCV, in particular, led to heightened sensitivity of latently infected cells to exogenous type I IFN. Through sequence analysis and reporter assays, we show that targets of these viral miRNAs include transcripts encoding the type I IFN receptors (IFNAR1, IFNAR2) and core components of JAK/STAT signaling pathways (JAK1, IRF9). Taken together, these data demonstrate that suppression of type I IFN responses is a conserved function for NHP γ-herpesvirus miRNAs and provide important mechanistic insight into how these viral miRNAs regulate type I IFN signaling pathways.

**IMPORTANCE:**

Gamma-herpesviruses establish life-long infections in their hosts. Evading anti-viral responses is a key component of long-term viral persistence. In this work, we show that small noncoding RNAs expressed by multiple non-human primate γ-herpesviruses regulate anti-viral responses by directly targeting components of the type I interferon (IFN) signaling pathway.

## INTRODUCTION

Human γ-herpesviruses, such as Epstein-Barr virus (EBV) and Kaposi’s sarcoma-associated herpesvirus (KSHV), are ubiquitous viruses that establish life-long, persistent infections and are linked to various cancers and inflammatory diseases, particularly in immunosuppressed individuals. KSHV is implicated in AIDS-related malignancies, such as Kaposi’s sarcoma and primary effusion lymphoma, as well as multicentric Castleman’s disease and KSHV inflammatory cytokine syndrome (1–3). EBV infection is linked to several types of lymphoma, nasopharyngeal carcinoma, gastric carcinoma, post-transplant disorders, as well as systemic autoimmune diseases, multiple sclerosis (MS), and most recently, long COVID (4–6).

A notable aspect of persistent γ-herpesvirus infection is the ability of these viruses to evade host anti-viral defense mechanisms. Both EBV and KSHV produce numerous viral microRNAs (miRNAs) that post-transcriptionally regulate gene expression and modulate several biological processes, including the activation of innate and adaptive immune responses (7–11). The exact mechanisms by which these viral miRNAs shape immunological responses are not completely elucidated; however, multiple studies demonstrate that human γ-herpesvirus miRNAs directly attenuate pattern recognition receptors (PRRs), interferon (IFN) signaling, cytokine production, and the subsequent signaling responses, which can impact T cell functions (9, 11–13). Specific examples of these activities include EBV miR-BART15-3p, which inhibits inflammasome activation and IL-1beta production by targeting NLRP3 (14). EBV miR-BART20-5p targets TBX21/Tbet, which decreases production of type 2 helper T cell (Th2) cytokines and IFN-gamma (15). EBV miR-BHRF1-2 blocks IL-1 signaling in infected B cells by reducing expression of the IL-1 receptor (16). IL12B, encoding the IL-12 cytokine that induces CD4+ T cell differentiation into type 1 helper T cells (Th1), and TAP2, critically involved in MHC I-mediated antigen presentation for CD8+ T cell activation, are targeted by multiple EBV miRNAs (10). *In vivo* experiments in humanized NOD/SCID mice with miRNA-deficient EBVs revealed key roles for viral miRNAs in attenuating the CD4+ and CD8+ T cell responses that control long-term latent infection (12).

Among the first line of defense against primary viral infections are type I IFNs produced by the innate immune system. Early detection of viral gene products by PRRs induces expression of IFNs, primarily IFN-alpha and IFN-beta, which are secreted and in turn, stimulate autocrine and paracrine signaling through type I IFN receptors (IFNARs). This activity subsequently induces anti-viral states in both infected and neighboring cells and is regulated on multiple levels. With regard to the human γ-herpesvirus miRNAs, several directly target nucleic acid sensors that trigger IFN production or target the core components of IFN signaling pathways, thereby inhibiting the responses to type I IFN. For example, the RIG-I/DDX58 3′UTR is targeted by both EBV miR-BART3 and miR-BART6-3p (8, 17). At least four KSHV miRNAs target the STING1 mRNA, thereby impairing cGAS/STING-mediated IRF3 phosphorylation, expression of IFN-beta, and interferon-stimulated gene (ISG) induction in response to infection (11). EBV miR-BART16 suppresses the transcriptional coactivator CBP (CREB binding protein), limiting induction of ISG transcripts (8, 18, 19), and KSHV miR-K12-11 inhibits IKK-epsilon expression, limiting IRF3 phosphorylation and ISG production (20). Downstream of IFNARs, components of the JAK/STAT pathways are regulated by multiple γ-herpesvirus miRNAs. KSHV miRNAs suppress IFN-alpha mediated activation of STAT3, subsequently reducing ISG levels in cells upon IFN treatment (21). EBV miR-BART8 can block expression of STAT1 (22), whereas miR-BART1 directly targets IRF9 (8). Additional components of IFN pathways, such as MX1, OAS proteins, and IRFs, are downregulated in response to KSHV miR-K12-1 (13). Moreover, EBV miR-BART1 targets the 3′UTR of SP100 (23), an IFN-stimulated nuclear factor involved in limiting replication of many different viruses, whereas EBV miR-BART5 targets the 3′UTR of ZCCHC3, a cGAS co-sensor that supports recognition of cytosolic dsDNA during viral infection (8, 24).

Rhesus lymphocryptovirus (rLCV) and rhesus macaque rhadinovirus (RRV) are simian counterparts of EBV and KSHV, respectively, and are linked to viral malignancies, such as lymphoma, in rhesus macaque (RM) models (25–27). Another non-human primate (NHP) rhadinovirus, Japanese macaque rhadinovirus (JMRV), is associated with an MS-like, inflammatory, demyelinating disease in Japanese macaques (28). Importantly, studies with these models have and continue to provide essential and unprecedented insight into viral dynamics, virus-driven immune modulation, and virus-associated pathologies (26, 29, 30). Akin to EBV and KSHV, these three NHP γ-herpesviruses encode numerous viral miRNAs, which are predicted to function as immune modulators similar to the human viral miRNAs. rLCV encodes a striking 35 different precursor (pre-) miRNAs (31–33), and homologous to EBV, these miRNAs are located in the rBHRF1 and rBART regions of the viral genome. The rLCV and EBV miRNAs are highly conserved; of the 25 EBV pre-miRNAs, 22 are conserved in rLCV (31–33). RRV and JMRV each encode 15 pre-miRNAs (34–36); homology to the KSHV miRNAs is limited to only a few miRNAs, although all RRV, JMRV, and KSHV miRNAs are located in conserved genomic positions (34–36).

Exact functions for RRV miRNAs are currently unknown; however, these viral miRNAs are detectable in RM cancers, such as retroperitoneal fibromatosis and RRV-associated B cell lymphoma (36). Only a select few RRV miRNAs exhibit sequence similarities to the KSHV miRNAs, although all RRV miRNAs do show high sequence homology to the JMRV miRNAs (34). In previous studies, we demonstrated that several JMRV miRNAs, such as miR-J8 and miR-J13, can attenuate nuclear factor kappa B (NF-kB) responses induced by pro-inflammatory cytokine activity (34). Comparative sequence analysis revealed that several RRV and JMRV miRNAs exhibit seed sequence homology to host miRNAs, such as members of the miR-17 family (i.e., miR-17-5p and miR-373), which target a variety of cellular transcripts, including those encoding components of the IFN signaling pathways. miR-17 can inhibit IRF9 in the context of herpes simplex virus 1 infection, whereas miR-373 targets both the JAK1 and IRF9 3′UTRs and blocks responses to type I IFNs (8, 37, 38). Notably, two rLCV miRNAs, miR-rL1-5 and miR-rL1-6, have high sequence homology to EBV miR-BART3 and miR-BART1, which both limit JAK/STAT signaling and can target JAK1 and IRF9 (8). These prior observations led us to hypothesize that NHP γ-herpesvirus miRNAs likely suppress IFN-mediated anti-viral responses and directly target components of the type I IFN pathway.

In this study, we sought to determine whether NHP γ-herpesvirus miRNAs could regulate cellular responses to type I IFNs. Using functional assays with miRNA expression vectors, as well as rLCV miRNA mutant viruses, we demonstrate that multiple viral miRNAs suppress transcriptional responses to type I IFN. Moreover, we show that several NHP γ-herpesvirus miRNAs directly target the 3′UTRs of IFNAR1 and IFNAR2, receptors that initiate the IFN signaling cascade, as well as core components of the JAK/STAT signaling pathway.

## RESULTS

### Functional screens identify multiple NHP γ-herpesvirus miRNAs that attenuate responses to type I IFN

**Fig 1:**
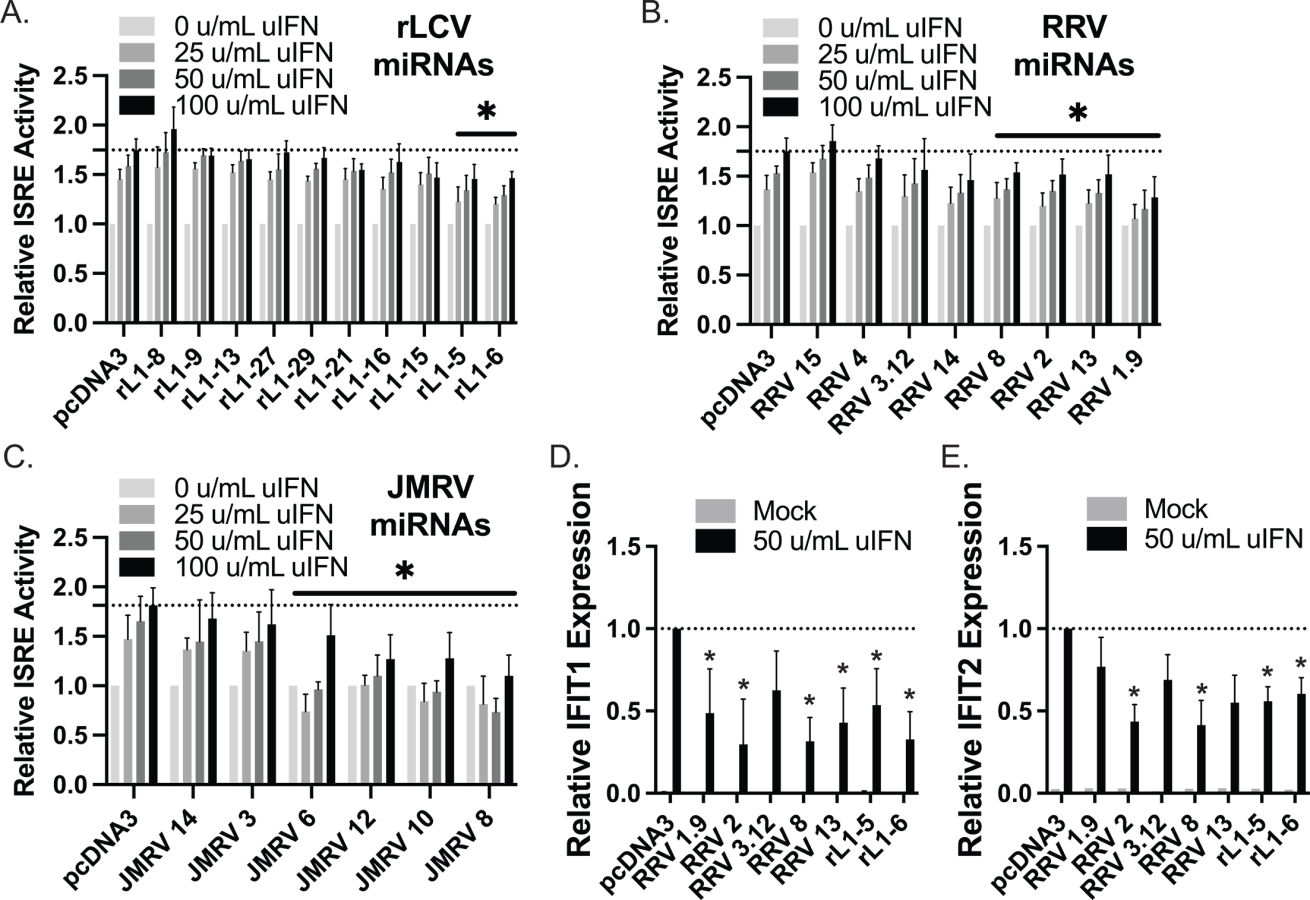
Screen of NHP γ-herpesvirus miRNAs for regulators of IFN signaling. (**A–C**) 293-ISRE cells were transfected with the indicated rLCV, RRV, or JMRV miRNA expression vectors. Forty-two hours post-transfection, cells were treated for 6 h with universal IFN (uIFN) to activate the ISRE reporter. Lysates were subsequently assayed for firefly luciferase activity. Values are normalized to mock-treated cells for each miRNA and reported relative to cells transfected with the control vector (pcDNA3). Graphs show the averages of at least six biological replicates for each miRNA vector. Dose responses were calculated as the area under the curve (AUC). *By *t*-test, *P* < 0.05. RLU = relative light units, representing fold activation over mock. (**D and E**) IFIT1 and IFIT2 expression are impacted by γ-herpesvirus miRNAs. 293T cells were transfected with the indicated rLCV or RRV miRNA expression vectors. Forty-two hours post-transfection, cells were treated for 6 h with 50 u/mL uIFN. Total RNA was assayed by qRT-PCR. Shown is the average of three experiments for IFIT1 and two experiments for IFIT2. Values are normalized to GAPDH and reported relative to cells transfected with the control vector (pcDNA3). *By Student’s *t*-test, *P* < 0.05.

To test the hypothesis that specific NHP γ-herpesvirus miRNAs block responses to type I IFN, we developed panels of viral miRNA expression vectors that included 10 RRV miRNAs, six JMRV miRNAs, and 10 rLCV miRNAs. Two of the RRV miRNA vectors encoded more than one pre-miRNA (i.e., RRV3.12 included miR-rR1-3 and miR-rR1-12, RRV1.9 included miR-rR1-1 and miR-rR1-9) while all other vectors encoded single pre-miRNAs. Among the RRV and JMRV miRNAs selected, we focused on ones that were abundantly detected in the context of primary infection in previous studies and included RRV miR-rR1-8 and JMRV miR-J8, which both exhibit seed homology to the cellular miR-17 family, as well as RRV miR-rR1-13 and JMRV miR-J13, which both exhibit seed sequence homology to miR-363/367 (34). The 10 rLCV miRNAs were selected based on their homology to EBV miRNAs, except for miR-rL1-21, which is rLCV-specific (32, 33). Using the panel of viral miRNA vectors, we then performed *in vitro* screens. Human 293-ISRE cells harboring an IFN-responsive firefly luciferase reporter were transfected with individual miRNA vectors, and after 42 h, treated for 6 h with increasing doses of IFN. We observed a nearly sixfold increase in ISRE activity over background in cells transfected with pcDNA3 control vector upon treatment with IFN (Fig. 1A through C). Consistent with phenotypes observed for EBV miR-BART1 and miR-BART3 (8), expression of the two rLCV homologs (rL1-6 and rL1-5) resulted in reduced activation of the ISRE reporter compared to pcDNA3 control cells (Fig. 1A). We also observed significantly lower IFN-mediated ISRE activation for four of the eight RRV miRNA vectors and four of the six JMRV miRNA vectors (Fig. 1B and C), showing that viral miRNAs from multiple NHP γ-herpesviruses suppress type I IFN responses. Notably, ectopic expression of RRV8 (miR-rR1-8) and JMRV8 (miR-J8), which exhibit sequence homology to miR-17 and miR-373, attenuated reporter activity in response to exogenous IFN.

Based on the results of the initial ISRE screen, we selected five RRV miRNA vectors and two rLCV miRNAs (both homologs of EBV miR-BART1 and miR-BART3) that significantly inhibited IFN-mediated ISRE activation for further investigation. RRV3.12 was included as the corresponding JMRV miRNA (miR-J12) significantly blocked activation of the ISRE reporter (Fig. 1C). Viral miRNA vectors were transfected into 293T cells for 42 h, cells were treated with IFN, and total RNA was harvested at 6 h to assess gene expression changes. qRT-PCR analysis of two different ISGs, IFIT1 and IFIT2, confirmed the suppressive effects of rLCV miR-rL1-5 and miR-rL1-6 on IFN responses (Fig. 1D and E). We further observed decreases in ISG induction in the presence of RRV miR-rR1-2, miR-rR1-8, and miR-rR1-13, which is consistent with results from the ISRE reporter assays. Interestingly, RRV3.12, which suppressed ISRE activation and expressed both miR-rR1-3 and miR-rR1-12, exhibited a partial, but not significant, reduction in ISG induction (Fig. 1D and E). As JMRV miR-J12 but not miR-J3 reduced ISRE activity (Fig. 1C), it is likely that only RRV miR-rR1-12 (homolog of JMRV miR-J12) functions to inhibit IFN responses and that these effects are masked in the context of the ISG assay when both RRV miRNAs are produced. Taken together, these data show that multiple NHP γ-herpesvirus miRNAs functionally inhibit type I IFN responses, including the transcriptional activation of ISGs.

### Viral miRNAs block type I IFN responses in rhesus fibroblasts

**Fig 2:**
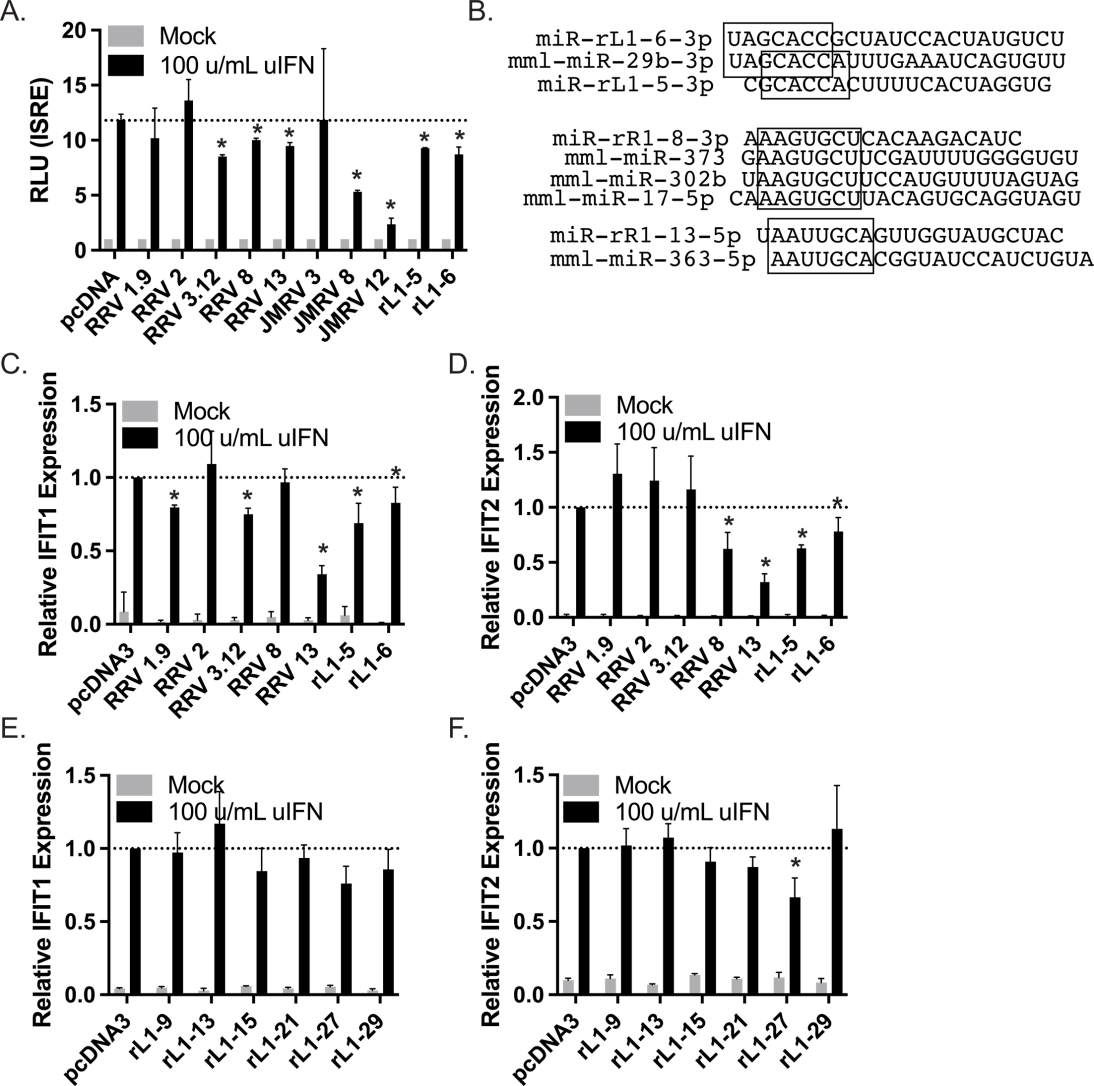
Viral miRNAs limit IFN-mediated ISG induction in tRFs. (**A**) tRF-ISRE cells were transfected with the indicated RRV, JMRV, or rLCV miRNA expression vectors. Forty-two hours post-transfection, cells were treated for 6 h with 100 u/mL uIFN. Lysates were harvested and assayed for firefly luciferase activity. Values are normalized to mock-treated cells for each miRNA and reported relative to cells transfected with the control vector (pcDNA3). Shown are the averages of three experiments. *By Student’s *t*-test, *P* < 0.05. (**B**) Sequence alignments of select RRV and rLCV miRNAs that exhibit seed homology to cellular miRNAs. (**C–F**) tRFs were transfected with the indicated RRV or rLCV miRNA expression vectors. Sixty hours post-transfection, cells were treated for 6 h with 100 u/mL uIFN. Total RNA was harvested and gene expression assayed by qRT-PCR. Shown are the averages of three experiments. Values are normalized to GAPDH and reported relative to cells transfected with the control vector (pcDNA3). *By Student’s *t*-test, *P* < 0.05.

Given the genetic differences in human versus rhesus macaque backgrounds, which could impact viral miRNA activity, we next tested IFN-related phenotypes for RRV, JMRV, and rLCV miRNAs in telomerase-immortalized rhesus fibroblasts (tRFs). tRF-ISRE cells were transfected with 10 different miRNA vectors and treated with IFN to activate the ISRE luciferase reporter. Similar to observations in 293 cells, ectopic expression of multiple viral miRNAs attenuated responses to type I IFN (Fig. 2A). Specifically, we observed significantly reduced ISRE activation in the presence of RRV miRNAs, such as miR-rR1-8 and miR-rR1-13, two JMRV miRNAs, miR-J8 and miR-J12, and two rLCV miRNAs, miR-rL1-5 and miR-rL1-6. It is worth noting that these specific viral miRNAs harbor seed sequence similarities to cellular miRNAs (Fig. 2B) with known functions in innate immunity and direct targets within IFN signaling pathways (8, 37–39).

To investigate further, we measured IFIT1 and IFIT2 induction in tRF cells transfected with the individual viral miRNAs. Both rLCV miR-rL1-5 and miR-rL1-6 limited ISG expression in response to IFN (Fig. 2C and D). Significant reductions in IFIT1 and IFIT2 were also observed for RRV miR-rR1-13 (Fig. 2C and D). Interestingly, ISG levels varied in the presence of RRV1.9, RRV3.12, and RRV8 miRNA vectors, which may be due to differences in the regulatory mechanisms responsible for IFIT1 and IFIT2 mRNA induction and stability. Evaluation of six additional rLCV miRNAs revealed trends in lower IFIT1 induction and significantly lower IFIT2 induction only in the presence of miR-rL1-27 (Fig. 2E and F). While viral miRNA phenotypes in tRFs were mostly consistent with those seen in 293 cells (Fig. 1), there were some minor inconsistencies that might be explained by genetic differences or the lower miRNA expression levels that were achievable in more difficult-to-transfect tRFs (Fig. S1). Overall, these results confirm that multiple NHP γ-herpesvirus miRNAs act to suppress IFN signaling.

### rLCV infection triggers ISG expression in PBMCs

**Fig 3:**
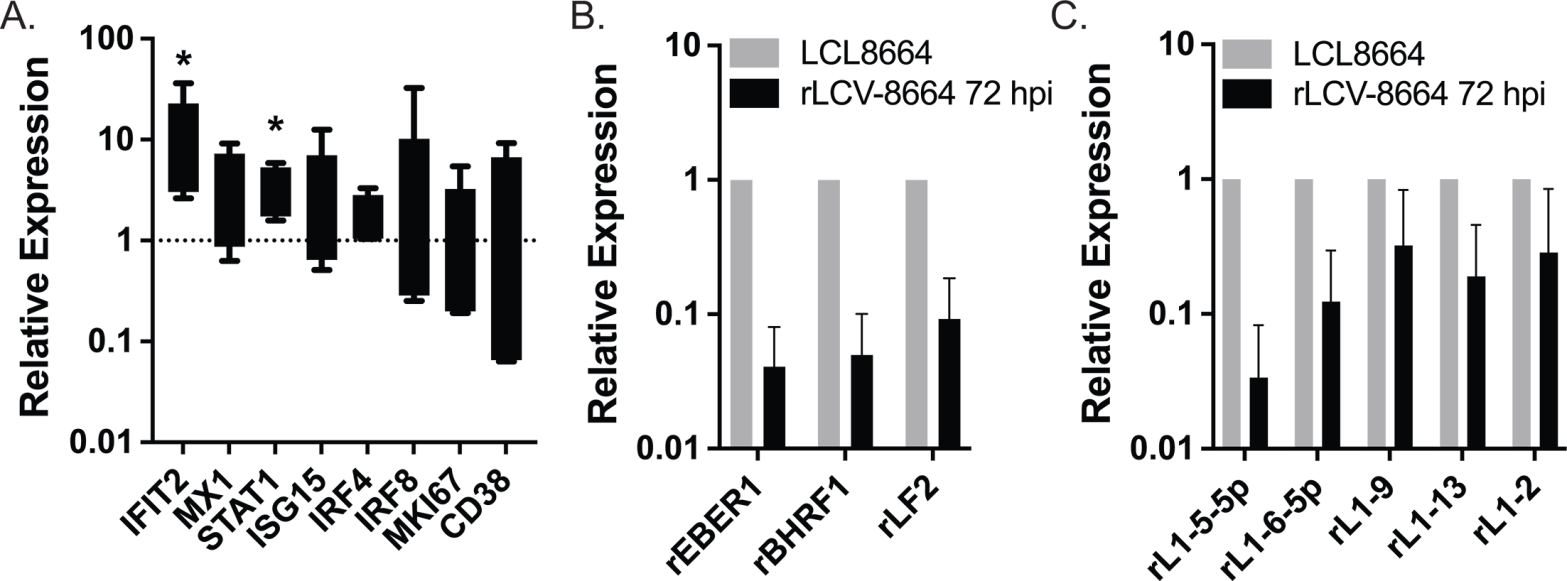
Cellular responses to *de novo* rLCV infection. (**A**) rLCV infection induces IFIT2 and STAT1 transcripts in PBMCs. PBMCs from rLCV-naïve RM donors were infected with wild-type rLCV (strain 8664). Total RNA was isolated at 72 hpi and tested for the indicated cellular genes. Values are normalized to GAPDH and reported relative to uninfected PBMCs. *By Student’s *t*-test, *P* < 0.05. Shown are the averages of five RM donors (donors 0, 10, 11, 3, and 4) with standard deviations. (**B**) rLCV gene expression at 72 hpi. No rLCV transcripts were detected in uninfected PBMCs. Values are normalized to GAPDH and reported relative to expression levels in LCL8664 cells. *n* = 4 donors. (**C**) rLCV miRNA expression at 72 hpi. Values are normalized to miR-16 and reported relative to expression levels in LCL8664 cells. Shown are the averages of four RM donors (donors 10, 11, 3, and 4) with standard deviations.

IFN production and the induction of ISGs have pivotal roles during the early stages of herpesvirus infections.  For EBV, overcoming this initial anti-viral response is thought to be indispensable for the establishment of latent infection, and deficiencies in controlling these early responses are potentially detrimental to long-term infection. As innate anti-viral responses have not been fully tested for rLCV infections *in vitro*, we investigated these initial responses in primary cells. RM peripheral blood mononuclear cells (PBMCs) from rLCV-seronegative RM were infected with the rLCV 8664 strain for 72 h. Total RNA was collected and assayed for cellular transcripts that included three ISGs (IFIT2, MX1, ISG15), as well as factors involved in B cell proliferation and differentiation (MKI67, IRF4, IRF8, CD38). Compared to uninfected cells, we observed significantly increased levels of IFIT2 and STAT1, as well as modest increases in IRF4, MX1, and ISG15 upon rLCV infection (Fig. 3A). In parallel, we assessed expression of three rLCV genes, rBHRF1, rEBER1, and rLF2, to determine their levels at 72 hpi (Fig. 3B). rEBER1 and rLF2 were selected since the EBV homologs of these genes have potential, albeit controversial, functions in activating pattern recognition receptors, such as RIG-I (8, 40). We also measured expression of five rLCV miRNAs representing homologs of the BHRF1 miRNAs (miR-rL1-2) and BART miRNAs (miR-rL1-5, -6, -9, -13) (Fig. 3C). Levels of the rLCV transcripts and viral miRNAs were detected at ~10% of the levels present in the latently infected LCL8664 cell line, which is expected given that B cells, the target cell type for infection, represent between ~5% and 15% of the PBMC population. Together, these results demonstrate that rLCV miRNAs are indeed produced in early-stage infection when ISGs are activated.

### rLCV miRNA mutant viruses exhibit heightened ISG signatures during primary infection

**Fig 4:**
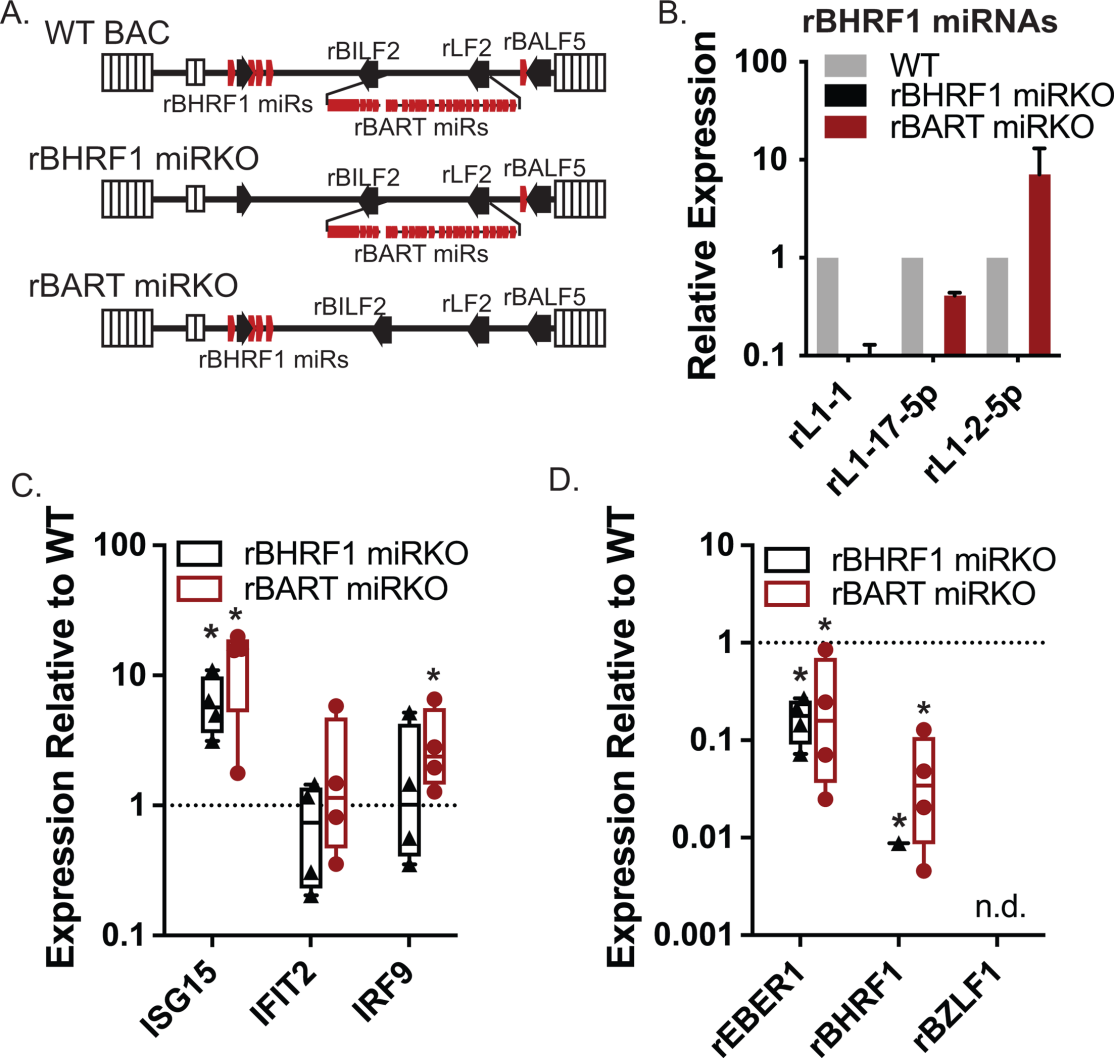
rLCV miRNAs impact ISG levels in primary infection. (**A**) Schematic of miRNA mutant rLCVs lacking either the rBHRF1 or rBART miRNA clusters. (**B**) rBHRF1 miRNA expression at 72 hpi. RM PBMCs were infected with P3HR1-derived WT, rBHRF1 miRKO, or rBART miRKO viruses (MOI = 200 genome equivalents/cell). Total RNA was harvested and miRNA levels evaluated by qRT-PCR. Values are normalized to miR-16 and reported relative to infections with WT virus. (**C**) ISGs are increased during infection with rLCV miRKO viruses. Same as B, but samples were tested for the indicated cellular transcripts by qRT-PCR. (**D**) rLCV gene expression in RM PBMCs infected with WT or miRKO viruses. Same as B, but samples were tested for the indicated genes. In (**C and D**), values are normalized to GAPDH and reported relative to infections with WT virus. Shown are the averages of four RM donors (donors 1, 2, 3, and 4) with standard deviations. *By Student’s *t*-test, *P* < 0.05.

**Fig 5:**
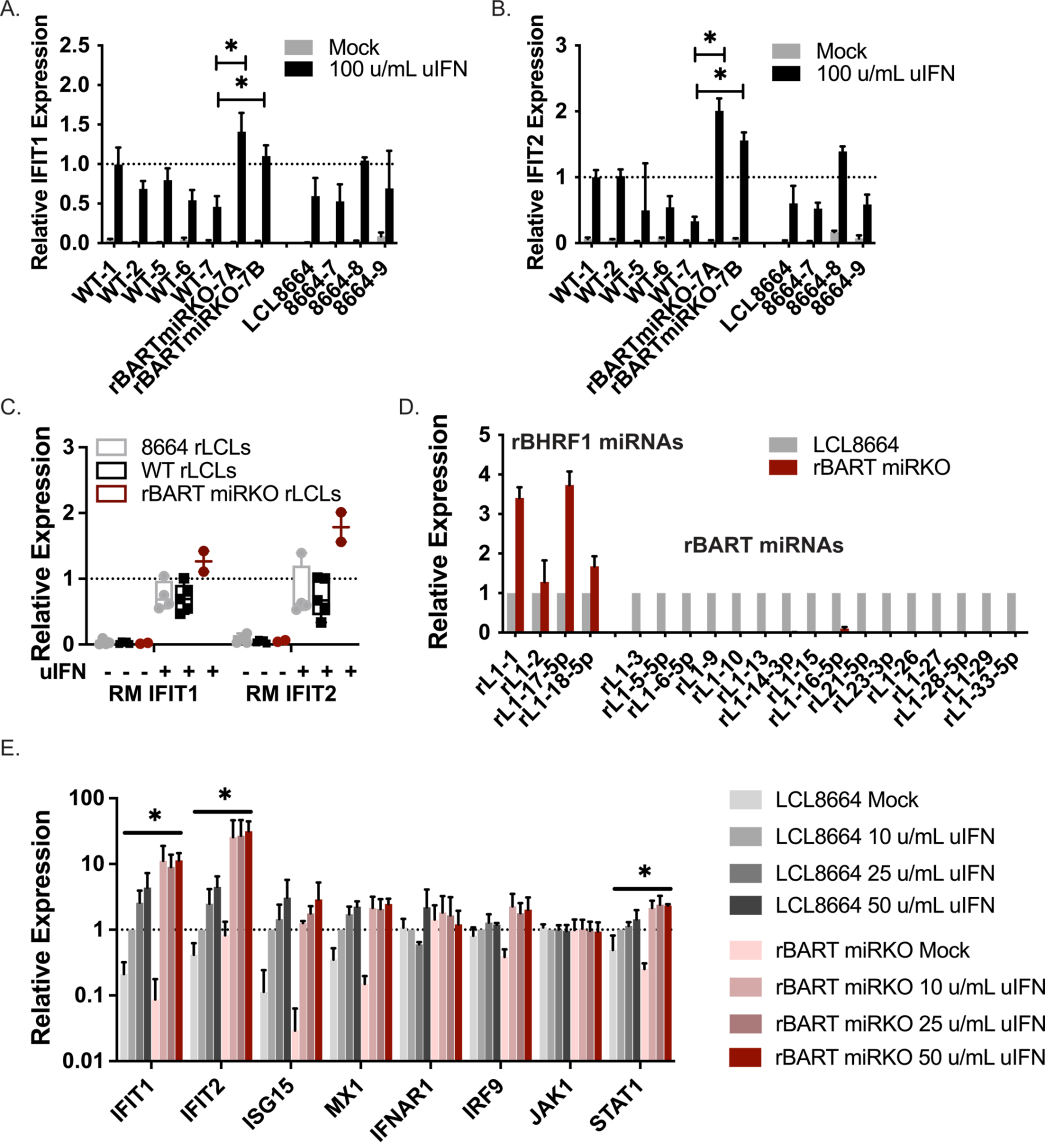
Viral miRNAs limit IFN-mediated ISG induction in latently infected cells. (**A and B**) Latently infected rLCLs established with rBART miRKO viruses exhibit heightened ISG responses to ectopic IFN. WT, rBART miRKO, and 8664 rLCLs were treated with 100 u/mL uIFN for 6 h. Total RNA was harvested, and IFIT1 and IFIT2 transcripts were evaluated by qRT-PCR. Values are normalized to GAPDH and reported relative to the response in WT-1 rLCL (donor 1 generated with WT recombinant rLCV). RM PBMC donors are indicated numerically. P3HR1-derived virus was used to generate WT-1 and WT-2 (MOI = 200), as well as WT-5 and WT-6 (MOI = 2,000). C33A-derived viruses were used to generate WT-7 and rBART miRKO-7A and -7B (MOI = 200). Shown are the averages of three biological replicates with standard deviations. *By Student’s *t*-test, *P* < 0.0.5. (**C**) Box and whisker plot of IFIT1 and IFIT2 expression in WT, 8664, and rBART miRKO rLCLs from **A and B**. Each point represents the average expression per donor. (**D**) rLCV miRNA expression in rLCLs established with rBART miRKO virus (donor 7). Total RNA was harvested and miRNA levels evaluated by qRT-PCR. Values are normalized to miR-16 and reported relative to levels in LCL8664. (**E**) rBART miRKO rLCLs and LCL8664 were treated with increasing doses of uIFN for 18 h. Total RNA was harvested and cellular transcripts evaluated by qRT-PCR. Values are normalized to GAPDH and reported relative to levels in mock-treated LCL8664. Shown are the averages of at least three biological replicates with standard deviations. Dose responses were calculated as AUC. *By *t*-test, *P* < 0.05.

To investigate functions of rLCV miRNAs during primary infection and ascertain whether these molecules influence IFN signaling in infected cells, we generated two rLCV recombinant viruses devoid of either the rBART miRNA or the rBHRF1 miRNAs. Briefly, *en passant* mutagenesis was performed using the WT rLCV bacterial artificial chromosome (BAC) backbone (based on the LCL8664 strain) (41). Mutations were designed in each of the rLCV pre-miRNA hairpins to disrupt seed sequences and predicted secondary structures, while preserving the rest of the RNA sequences. Destabilized hairpin structures were confirmed by the lack of appropriate folding using the RNAfold Web Server, and <10 predicted targets for each mutated miRNA seed sequence were determined according to TargetScan Custom v5.2. For the rBHRF1 miRNA mutant, the entire region was synthesized, cloned into an expression vector, and the lack of viral miRNA production was confirmed in 293T cells (Fig. S2C) prior to introducing this region into the rLCV BAC. BAC mutants were confirmed by three methods: (i) sequencing of PCR products amplified from the rLCV BART and BHRF1 regions, (ii) restriction enzyme digestion, followed by gel electrophoresis to confirm intact BAC DNAs (Fig. S2A and B), and (iii) loss of miRNA expression in infected cells (see Fig. 4 and 5). To generate infectious virus, rLCV BAC DNAs were introduced by electroporation into P3HR1 cells, and the cells were placed under hygromycin selection. Virus was subsequently purified from the supernatant of hygromycin-resistant P3HR1 producer cells induced for lytic replication, quantified by qPCR for rLCV genome copies, and used to infect RM PBMCs.

To investigate responses in early infection, RM PBMCs from four rLCV-naïve RM donors were infected with WT, rBHRF1 miRKO, or rBART miRKO recombinant viruses (MOI = 200 genome equivalents per cell). At 72 hpi, RNA was isolated, and three ISGs (ISG15, IFIT2, IRF9) were tested by qRT-PCR. rBHRF1 miRNA expression was confirmed in WT and rBART miRKO infections (Fig. 4B). Compared to infection with the WT virus, significantly heightened levels of ISG15 and IRF9 were observed upon infection with the rBART miRKO virus (Fig. 4C). We also observed modest upregulation of IFIT2 in the absence of the rLCV BART miRNAs. ISG15 was significantly upregulated in cells infected with the rBHRF1 miRKO virus; however, no statistically significant changes in IFIT2 or IRF9 were detected. To evaluate infectivity, we measured three different viral transcripts. rBZLF1 was undetectable for any sample, suggesting that observed phenotypes are not associated with an increase in lytic replication (Fig. 4D). Interestingly, reduced rEBER1 and rBHRF1 levels were observed for both of the rLCV miRNA mutant viruses (Fig. 4D), suggesting a potentially lower level of infection that is concurrent with increased innate immune activity in the absence of the rLCV miRNAs. These data further indicate that, while loss of either the rBHRF1 or rBART miRNAs leads to heightened ISG activity, it is the rBART miRNAs that appear to play a more substantial role in mediating these responses.

### rLCLs latently infected with rBART miRKO viruses exhibit increased sensitivity to IFN

To investigate whether rLCV miRNAs modulate IFN responses during latent infection, we established rLCLs with rBART miRKO virus or control viruses harboring intact rBART miRNA regions (i.e., WT and 8664 strains). rBART miRNA expression—specifically lack thereof in the mutant—was confirmed by qRT-PCR in resultant rLCLs (Fig. 5D). rBART miRKO rLCLs were positive for rEBER1 (latent marker) and expressed low levels of two lytic genes (rBHRF1 and rBZLF1) that were in the range of rLCLs harboring either WT or 8664 viruses (Fig. S3). To evaluate IFN sensitivity, we initially treated rLCLs with 100 u/mL IFN for 6 h and tested two ISGs (IFIT1 and IFIT2). Compared to LCL8664 cells and eight other WT or 8664-derived rLCLs, two independently derived rBART miRKO rLCLs exhibited heightened levels of both IFIT1 and IFIT2 in response to exogenous IFN (Fig. 5A through C). As the donor matched WT-7 rLCL showed the lowest level of ISG induction compared to other rLCLs, we used LCL8664 as a control for dose response experiments and evaluated additional ISGs. Cells were treated with increasing doses of IFN for 18 h. Significant increases in both IFIT1 and IFIT2 were observed in rBART miRKO rLCLs relative to LCL8664 (Fig. 5E). We also observed significant increases in STAT1 expression in rBART miRKO rLCLs compared to LCL8664 (Fig. 5E), which could be due to the lower basal level of STAT1 in untreated rBART miRKO cells. In contrast, quantification of other cellular transcripts (MX1, IFNAR1, or JAK1) by qRT-PCR showed no significant differences (Fig. 5E). Collectively, these data provide evidence that the rLCV rBART miRNAs modulate type I IFN-mediated transcriptional responses in the context of viral infection and likely confer resistance to anti-viral mechanisms that may inadvertently become activated during latency.

### rLCV miRNAs target major type I IFN signaling components in rLCLs

**Fig 6:**
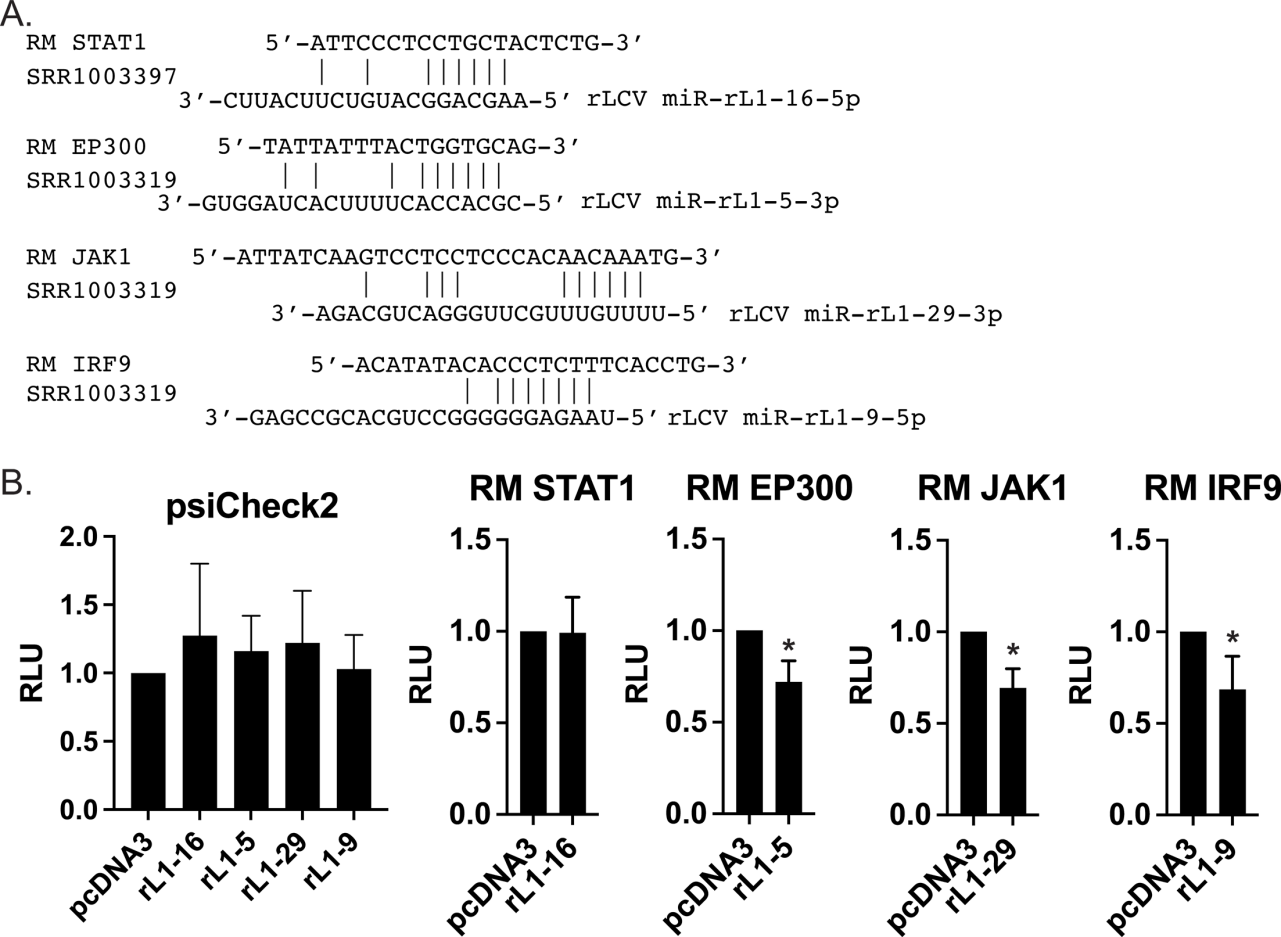
rLCV miRNAs target components of IFN signaling pathways. (**A**) PAR-CLIP-identified Ago interaction sites for several rLCV miRNAs. Two published PAR-CLIP data sets from latently infected rLCLs were re-analyzed to define viral miRNA binding sites within cellular 3′UTRs. Shown are canonical miRNA interactions (≥7mer1A) between rLCV miRNAs and the RM 3′UTRs for STAT1, EP300, JAK1, and IRF9. (**B**) 3′UTR luciferase reporter assays confirm miRNA interactions. RM 3′UTRs of indicated genes were cloned into psiCheck2. 293T cells were co-transfected with 25 ng of luciferase reporter and 250 ng of miRNA expression vector. Lysates were harvested 48–72 h post-transfection and assayed for dual luciferase activity. Shown are the averages of at least three experiments in triplicate with standard deviations. Values are reported relative to pcDNA3 control vector. RLU, relative light units. *By Student’s *t*-test, *P* < 0.05.

To gain mechanistic insight into how rLCV miRNAs functionally inhibit the type I IFN pathway and identify direct targets for these miRNAs in infected cells, we re-analyzed two previously published Ago-PAR-CLIP data sets from rLCLs. While several viral transcripts regulated by rLCV miRNAs have been previously reported in these data sets, cellular targets have not been examined (33). Raw fastq files were pre-processed, aligned to the rhesus macaque genome (Mmul_10), and Ago interaction sites defined by PARalyzer (42). Ago binding sites specifically within the 3′UTRs of macaque protein-coding transcripts were determined and the corresponding genes analyzed using Reactome (43) to define targets associated with “Interferon Signaling” (R-HSA-913531) and “Interferon alpha/beta signaling” (R-HSA-909733). CLIP’ed sites in these 3′UTRs were then interrogated for canonical seed matches to the rLCV miRNAs. We focused on the rLCV BART miRNA homologs since BART miRKO viruses showed significant phenotypes in responding to type I IFN (Fig. 5). Through this analysis, we identified STAT1, JAK1, and IRF9, which are key components of the IFN signaling pathway, as rLCV miRNA targets (Fig. 6A). Additionally, EP300, a member of the p300/CBP family that is involved in transcriptional co-activation of STAT-responsive and IFN-responsive genes, was identified as a target of rLCV miR-rL1-5.

To confirm the identified interactions, we cloned the 3′UTRs of these four genes into psiCheck2. Reporters were co-transfected with the corresponding rLCV miRNA expression vectors, and luciferase activity was measured. Except for the STAT1 3′UTR, which did not respond to rLCV miR-rL1-16, we observed knockdown of luciferase expression for each reporter upon ectopic expression of the targeting rLCV miRNA (Fig. 6B). The RM JAK1 reporter was targeted by miR-rL1-29, whereas the RM IRF9 reporter was targeted by miR-rL1-9 (Fig. 6B). Notably, rLCV miR-rL1-5, which exhibited significant phenotypes in functional assays in both human 293 cells and tRFs (Fig. 1 and 2), inhibited the RM EP300 reporter (Fig. 6B). Thus, rLCV BART miRNA homologs target several components of type I IFN signaling, as well as a transcriptional co-activator of STAT-responsive genes in latently infected rLCLs.

### NHP γ-herpesvirus miRNAs target multiple core components of IFN signaling pathways

To identify other potential targets for rLCV, RRV, and JMRV miRNAs that substantially block IFN responses, we used bioinformatics approaches to predict viral miRNA binding sites. Initially, we used TargetScan Custom 5.2 (44) with “Rhesus” as the selected species to search for possible target genes harboring canonical seed matches to the viral miRNAs. Predicted target lists were subsequently analyzed with Reactome (43) to determine genes specifically associated with “Interferon alpha/beta signaling” (R-HSA-909733). Through this analysis, we identified IFNAR1, IFIT2, IFIT5, SLC7A8, and IRF2 as potential viral miRNA targets. As TargetScan Custom 5.2 incorporates evolutionary conservation constraints in evaluating putative miRNA binding sites, and viral miRNAs are not well conserved with host miRNAs (Fig. 2B; Table S1), we took one more approach. Rhesus macaque 3′UTR sequences for core components of the JAK/STAT signaling pathway (IFNAR1, IFNAR2, JAK1, TYK2, IRF9, STAT1, and STAT2) were downloaded from Ensembl and scanned directly for canonical miRNA seed match sites using RNAhybrid (45). With the exception of TYK2, binding sites for at least one NHP γ-herpesvirus miRNA in these 3′UTRs were identified.

**Fig 7:**
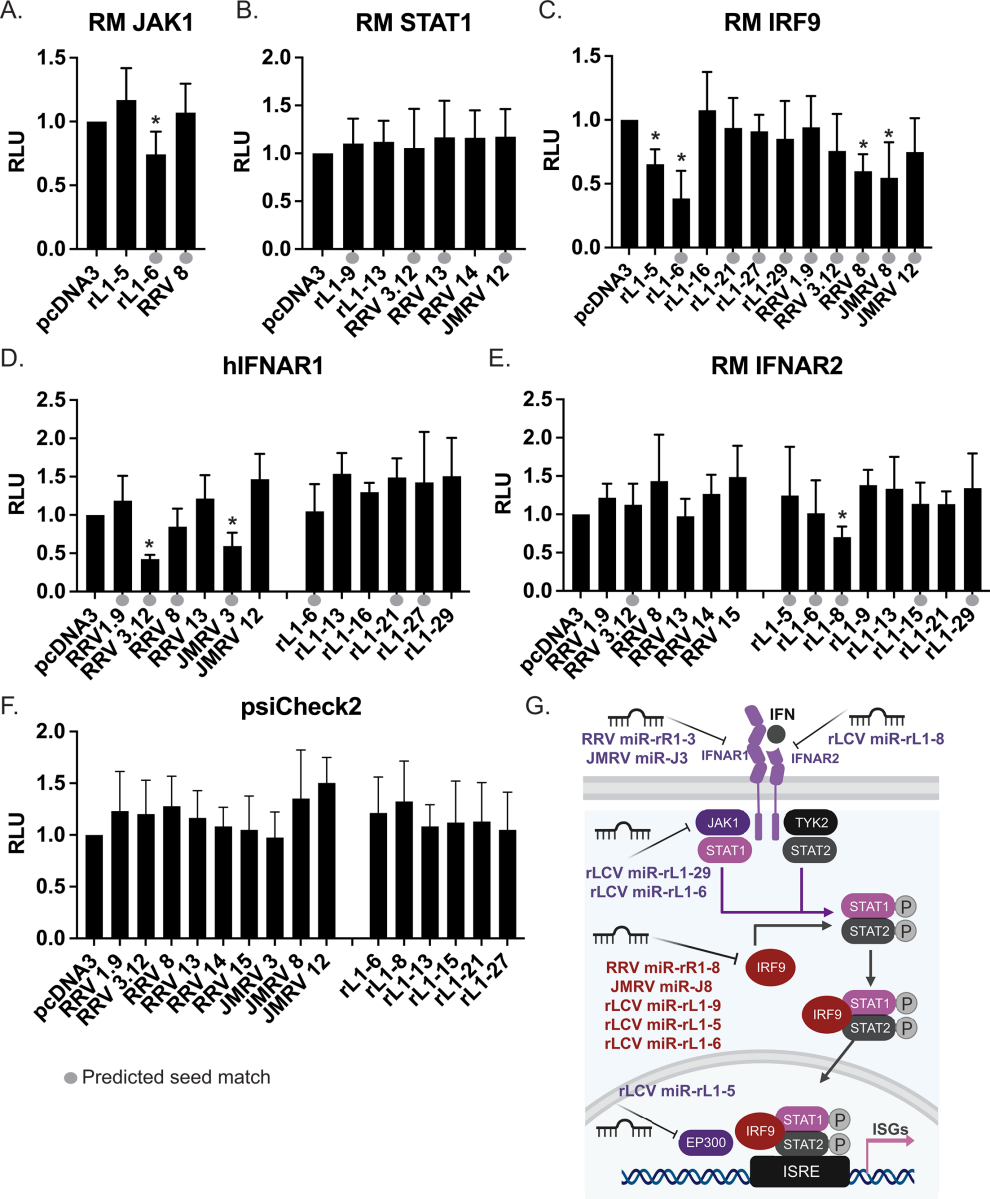
Validation of predicted viral miRNA binding sites by 3′UTR luciferase assays. (**A–F**) Evaluation of predicted viral miRNA interactions by 3′UTR luciferase reporter assays. RM 3′UTRs of indicated genes were cloned into psiCheck2. 293T cells were co-transfected with 20 ng of luciferase reporter and 250 ng of miRNA expression vector. Lysates were harvested 48–72 h post-transfection and assayed for dual luciferase activity. Shown are the averages of at least three experiments in triplicate with standard deviations. Values are reported relative to pcDNA3 control vector. Gray circles indicated a predicted seed match for the viral miRNA and corresponding 3′UTR. RLU, relative light units. *By Student’s *t*-test, *P* < 0.05. (**G**) Diagram of the type I IFN signaling pathway and the components that are targeted by NHP g-herpesvirus miRNAs.

To determine whether any of the predicted interactions were functional, we then tested the JAK1, STAT1, and IRF9 3′UTR reporters against additional viral miRNA vectors. Notably, rLCV miR-rL1-6 significantly knocked down the JAK1 reporter, whereas multiple viral miRNAs targeted the IRF9 reporter (Fig. 7A and C). Both rLCV miR-rL1-5 and miR-rL1-6, which have homology to EBV BART miRNAs, inhibited luciferase expression from the IRF9 reporter. Moreover, the miR-17/miR-373 seed homologs encoded by RRV and JMRV (miR-rR1-8 and miR-J8) suppressed activity of the IRF9 3′UTR. We formally confirmed that RRV miR-rR1-8 can target 3′UTRs with binding sites for miR-17 family members by testing this construct against a miR-17-5p luciferase indicator (Fig. S4). To test additional predicted interactions (Table S2), we next cloned the IFNAR2 3′UTR from rhesus cells and the IFNAR1 3′UTR from human 293T cells (this region has high sequence homology to rhesus). The hIFNAR1 reporter responded significantly to RRV3.12 and JMRV miR-J3 (Fig. 7D), whereas the RM IFNAR2 reporter responded to rLCV miR-rL1-8 (Fig. 7E), indicating these miRNAs likely block the initiation of the IFN signaling cascade by reducing expression of the IFN receptors. A summary of all confirmed interactions between specific IFN pathway components within the JAK1/STAT1/IRF9 signaling axis and viral miRNAs is shown in Fig. 7G.

Previous studies on EBV miRNAs have revealed interactions with cellular targets involved in other arms of innate immunity that are relevant to viral replication and signaling through type I IFN (8, 23). We therefore tested RM CHUK (Ikk-alpha) and IKBKB (Ikk-beta) 3′UTRs against rLCV miRNAs, as these were previously reported targets of the EBV BART miRNAs (8), and there is extensive cross-talk between the IFN and NF-kB signaling pathways (Fig. S4). We also tested three human 3′UTRs (DAZAP2, SP100, and ZCCHC3) which are confirmed targets of EBV miR-BART3, miR-BART1, and miR-BART5 (8, 23), homologs of rLCV miR-rL1-5, miR-rL1-6, and miR-rL1-8, respectively. The three conserved human genes encode innate immune factors involved in host anti-viral defenses that impact the replication of various viruses (24, 46, 47). IFN-inducible SP100, for example, is a component of promyelocytic leukemia bodies that restricts EBV replication in the absence of EBNA-LP (48). With the exception of IKBKB, ectopic rLCV miRNA expression inhibited luciferase activity from all the reporters (Fig. S4), validating these as rLCV miRNA targets. Taken together, these findings demonstrate that NHP γ-herpesvirus miRNAs regulate multiple host innate immune signaling components relevant to IFN activity and signaling responses.

## DISCUSSION

In this study, we demonstrate that viral miRNAs encoded by multiple NHP γ-herpesviruses commonly suppress type I IFN responses by directly targeting numerous components of the IFN signaling pathway. Specifically, rLCV miRNAs targeted the 3′UTRs of core type I IFN pathway components (IFNAR2, JAK1, and IRF9) and also targeted EP300, which co-activates STAT-mediated transcription. RRV and JMRV miRNAs targeted IFNAR1, as well as IRF9, which is required to drive ISG induction. Analysis of miRNA mutant rLCVs further revealed enhanced ISG activation in early phase infection and increased sensitivity of latently infected rLCLs lacking the rBART miRNAs to exogenous type I IFN. Such findings extend prior observations in human γ-herpesviruses and further highlight that suppression of IFN-mediated anti-viral mechanisms is a conserved strategy shared amongst primate herpesvirus miRNAs.

Our results align with earlier reports that EBV and KSHV miRNAs attenuate host anti-viral responses by disrupting nucleic acid sensing, type I IFN production, and IFN-mediated signal transduction (8, 11, 17, 18, 20). For example, EBV miR-BART16 suppresses CREB-binding protein, a member of the p300/CBP family that co-activates STAT-mediated transcription (18), KSHV miRNAs repress STING (11), and several EBV miRNAs directly target SP100, ZCCHC3, RIG-I, JAK1, and IRF9 (8, 17, 23). Identifying analogous mechanisms for suppressing anti-viral signals in NHP γ-herpesviruses indicates that these viral miRNA-mediated activities are under selective evolutionary pressure. Beyond direct components of IFN signaling, we also observed that rLCV miRNAs can target 3′UTRs of genes involved in DNA sensing and restricting viral replication (i.e., SP100, ZCCHC3, DAZAP2). The fact that these mechanisms are conserved by both lymphocryptoviruses and rhadinoviruses underscores the biological importance of viral miRNA activity in dampening innate immune responses to promote virus infection.

Importantly, our results show that rLCV BART miRNAs can target multiple levels of the type I IFN signaling pathway, from the cell-surface receptor (IFNAR2) level that initiates the signaling cascade to intermediate signaling components (JAK1, IRF9) and finally, to the level of transcriptional activation (EP300) of ISGs within the nucleus. NHP rhadinovirus miRNAs act in a similar manner—through luciferase reporter assays, we confirmed both IFNAR1 and IRF9 as RRV and JMRV miRNA targets. Interestingly, such redundancies in pathway regulation by viral miRNAs are observed for many herpesviruses. For instance, human cytomegalovirus (hCMV) miRNAs target functionally related genes within the secretory pathway to modulate cytokine release, as well as virion assembly during productive infection (49). Multiple EBV, KSHV, and hCMV miRNAs regulate various components of NF-kB signaling pathways to alter cytokine production, cell survival, and inflammation (16, 33, 50–52). We previously showed that rLCV and JMRV miRNAs also function to attenuate NF-kB signaling activated by either pro-inflammatory cytokines or directly through viral proteins (i.e., LMP1) (33, 34). Here, we further show evidence that one rLCV miRNA, miR-rL1-16, can even target the 3′UTR of CHUK (Ikk-alpha) (Fig. S4), which is a core component of the NF-kB cascade and also regulated by hCMV miRNAs (53). The multi-layer regulation of single components within a pathway can help to ensure that even if individual targets are only partially inhibited by a given viral miRNA, a pathway can still be effectively suppressed overall. Additionally, some components are targeted by more than one viral miRNA. In this study, for example, we show the IRF9 3′UTR can be targeted by rLCV miR-rL1-9, miR-rL1-5, and miR-rL1-6 (Fig. 6 and 7). Moreover, as common consequences of both IFN and NF-kB signaling are the transcriptional activation of anti-viral and pro-inflammatory genes, such as cytokines that recruit immune effector cells, our results support the growing body of evidence showing that herpesvirus miRNAs are important for immune evasion.

Our experiments utilized two different rLCV miRNA mutant viruses to start to address the separate contributions of the rBHRF1 and the rBART miRNA homologs in modulating innate immune responses during infection. Prior studies with EBV utilized viruses in which all viral miRNAs were deleted (8), thus the exact roles of these distinct miRNA clusters in the context of type I IFN signaling are not completely defined. Through evaluation of a handful of ISGs, we determined that infection with viruses lacking either of the rLCV miRNA clusters resulted in increased ISG15 levels compared to miRNA-intact virus, indicating that both rBHRF1 and rBART miRNA clusters can counteract ISG15 induction. Notably, however, there were observable differences in the two viruses. Viruses lacking the rBART miRNAs produced more substantial phenotypes than viruses lacking the rBHRF1 miRNAs, as significant increases in IFIT2 and IRF9 transcripts were observed only with rBART miRKO virus in early-stage infection (Fig. 4). We established rLCLs with rBART miRKO virus, demonstrating that this rLCV recombinant is indeed transforming (Fig. 5). Compared to rLCLs harboring an intact rBART region, rBART miRKO rLCLs exhibited increased sensitivity to exogenous type I IFN as determined by elevated induction of IFIT1, IFIT2, and STAT1. Such findings are consistent with the cellular targets identified for the rLCV BART miRNA homologs (Fig. 6 and 7) and the numerous EBV BART miRNAs that individually attenuate ISRE and STAT signaling in prior studies (8, 18). Importantly, these results further demonstrate that BART miRNAs continue to protect cells from immune responses during latent infection.

Beyond our *in vitro* findings that NHP γ-herpesvirus miRNAs directly interfere with IFN signaling, our work establishes the foundation for future studies aimed at investigating how these molecules contribute to immune regulation, viral persistence, and disease phenotypes *in vivo*. Herpesviruses encode numerous proteins that aid in immune evasion, and a plethora of *in vitro* studies show that most of these proteins are expressed in primary infection stages or following lytic reactivation. In the context of RRV infection, for example, virally encoded vIRFs antagonize type I and type II IFN signaling and delay the development of host immune responses following acute infection (54, 55); presumably, this supports the initial establishment of viral reservoirs for long-term persistence. During latent infection, however, viral gene expression is highly limited. A key advantage of viral miRNAs is that, unlike viral proteins, these molecules are non-immunogenic and thus capable of modulating immune responses without eliciting immune recognition. Thus, viral miRNA activity to disrupt activation of anti-viral responses, particularly during periods of latency, may support the maintenance of infected cells within a host.

In conclusion, our study demonstrates that direct impairment of type I IFN signaling via viral miRNAs is a common and conserved strategy utilized by γ-herpesviruses. Future investigations into elucidating the *in vivo* roles of these molecules will enhance our understanding of how herpesviruses evade the immune system and can lead to the development of new viral intervention strategies.

## MATERIALS AND METHODS

### Cell culture and transfections

293T and tRFs were maintained at 37°C in Dulbecco’s modified Eagle’s medium supplemented with 10% fetal bovine serum (FBS), penicillin, and streptomycin. LCL8664 (ATCC CRL-1805), rhesus LCLs, and P3HR1 cells were maintained at 37°C in RPMI supplemented with 15% FBS, penicillin, and streptomycin. 293-ISRE and tRF-ISRE reporter cells, maintained under puromycin selection, were generously provided by Dr. Victor DeFilippis’s lab at the VGTI and constitutively express an IFN-responsive firefly luciferase reporter. Cell lines were transfected using Lipofectamine 2000 (Life Technologies) according to the manufacturer’s protocol.

### Recombinant viruses and virus production

The recombinant rhesus LCV BAC was kindly provided by Dr. Fred Wang at Harvard Medical School and is described in reference 41. Mutations in the rLCV BHRF1 and BART miRNA-encoding regions were introduced using *en passant* mutagenesis. Oligonucleotides and gene blocks used to produce miRNA mutant viruses can be provided upon request. To confirm intact BACs, rLCV BAC DNAs were isolated from *Escherichia coli* using the NucleoBond BAC 100 kit (Macherey-Nagel), digested with restriction enzymes, and the correct banding patterns were confirmed by gel electrophoresis. To recover infectious viruses, BAC DNA was transfected into P3HR1 or EBV-negative C33A cells as previously described (41, 56), and hygromycin-resistant cultures were established. Virus reactivation for P3HR1 cells was induced by 50 ng/mL PMA treatment and 3 mM sodium butyrate for 24 h, followed by re-seeding in fresh media. Virus reactivation for C33A cells was induced by transfection of BZLF1 and BRLF1, followed by treatment with 50 ng/mL PMA and 3 mM sodium butyrate for 24 h (41). To generate virus from LCL8664, cells were treated with 50 ng/mL PMA and 3 mM sodium butyrate for 24 h and re-seeded in fresh media at 2 × 10^6^ cells/mL. Virus-containing supernatants were harvested after 5–7 days in culture. All cell-free supernatants were filtered through 0.45 uM filters and concentrated by centrifugation. Stocks were stored at −80°C. Viral genome copies were quantified in virus stocks by qPCR using primers against the rLCV IR1 region. Mutations in the miRNA-encoding loci were confirmed by PCR amplification of these regions and Sanger sequencing of PCR amplicons.

### rLCV infections *in vitro*

PBMCs were isolated from RM (rLCV-naïve) whole blood collected during routine screening for rLCV. Blood collection was conducted under a protocol approved by the Institutional Animal Care and Use Committee at OHSU, and the studies were carried out in strict accordance with the recommendations in the Guide for the Care and Use of Laboratory Animals. For infections, RM PBMCs were seeded into 24-well plates at 3–5 × 10^5^ cells/well in RPMI supplemented with 15% FBS, penicillin, streptomycin, and 400 ng/mL cyclosporin A. Virus was added to each well (MOI = 200 or 2,000 genome equivalents per cell as determined by IR1 qPCR). Media was replaced after 18–24 h, and cells were maintained at 37°C. All RM blood samples were pre-screened for rLCV using qPCR; only PBMCs testing negative for rLCV were used for assays. Outgrowing rhesus LCLs were determined microscopically and were passaged twice a week in RPMI supplemented with 15% FBS, penicillin, and streptomycin at 37°C. To test for EBV in RM PBMCs subject to infection with P3HR1-derived WT rLCV, two EBV-specific miRNAs were evaluated (Fig. S2D). To test for EBV in resultant rLCLs established with P3HR1-derived recombinant viruses, genomic DNA was isolated as previously described, and qPCR primers against the EBV LMP1 region were utilized to detect EBV genomes (57) (Fig. S2E).

### Viral miRNA expression vectors

Expression vectors for several rLCV and JMRV miRNAs are previously described (33, 34). To generate additional viral miRNA expression vectors, 150 to 450 nt surrounding a viral pre-miRNA or pairs of pre-miRNAs were PCR amplified from genomic DNA isolated from either RRV-infected RFs or rLCLs and cloned into the XhoI and XbaI sites of pcDNA3 as previously described (34). Oligonucleotide sequences used to generate miRNA vectors are provided in Table S1. To confirm miRNA expression, vectors were transfected into 293T cells, total RNA was harvested after 48 to 72 h, and miRNA levels were assayed by TaqMan qRT-PCR (Fig. S1). miR-16 or U6 was tested as an internal control. Functional activity for several viral miRNAs was additionally confirmed by luciferase reporter assays (Fig. S4).

### RNA isolation and qRT-PCR

Total RNA was isolated from cells using TRIzol (Thermo Fisher) according to the manufacturer’s protocol, except substituting the 70% ethanol wash step with 95% ethanol. To measure gene expression, RNA was DNase-treated and reverse transcribed using MultiScribe (Thermo Fisher) with random hexamers. Cellular and viral transcripts were detected using qPCR primers to gene-specific regions and PowerUp SYBR green qPCR master mix (Thermo Fisher). miRNAs were measured by TaqMan qRT-PCR assays, using miR-16 or U6 as internal controls. All qPCR reactions were performed in technical replicates, and relative quantities were calculated using the 2^−ΔΔCt^ method.

### Luciferase reporter assays

293-ISRE or tRF-ISRE cells were transfected with 250 ng pcDNA3 control or viral miRNA expression vectors in 96-well black-well plates. At 42 h post-transfection, cells were stimulated with indicated amounts of uIFN (25 units, 50 units, or 100 units/mL) for 6 h and subsequently lysed in 1× passive lysis buffer (Promega). Firefly luciferase reporter activity was measured on a luminometer following the addition of Luciferase Assay Reagent II (Promega). For 3′UTR reporter assays, 20 ng of psiCheck2-based vectors containing either the human or rhesus macaque 3′UTRs of indicated genes were co-transfected into 293T cells with 250 ng of pcDNA3-based miRNA expression vector. Cells were harvested at 48–72 h post-transfection and assayed for luciferase activity using the Dual Luciferase Assay kit (Promega).

### Bioinformatics analysis of Ago PAR-CLIP data sets from rLCLs

Raw sequencing reads for rLCL 211-98 (SRX360506/SRR1003319) and rLCL 309-98 (SRX360507/SRR1003397) PAR-CLIP data sets (NCBI BioProject PRJNA218007) were obtained in FASTQ format and pre-processed using Trim Galore (github.com/FelixKrueger/TrimGalore) to remove Illumina adapter sequences and reads that contained Ns or were <17 nt in length. Reads were aligned to the Macaca mulatta Mmul_10 genome (GCF_003339765.1) using Bowtie2 v2.5.3 with default parameters. PARalyzer v1.5 (42) was used to define Ago interaction sites, with the following parameters: minimum_readcount_per_cluster = 3, minimum_cluster_size = 17, and minimum_conversion_count_cluster = 1. Clusters were annotated by aligning back to Mmul_10 3′UTRs (Ensembl Genes v112) using Bowtie2 v2.5.3 with default parameters, and subsequently, 3′UTR sites were scanned for canonical miRNA seed matches (≥7mer1A) to the rLCV miRNAs.

### miRNA target prediction

rLCV, RRV, and JMRV miRNA targets were predicted based on canonical 5′ miRNA seed sequence matches to protein-coding cellular transcripts using TargetScan Custom v5.2 (44) and “rhesus macaque” as the selected species. As a second strategy, 3′UTR sequences for IFNAR1, IFNAR2, IRF9, JAK1, STAT1, STAT2, TYK2, and IFIT2 were obtained directly from Ensembl (rheMac10) and scanned for canonical seed-match sites (nt 2–7 pairing, no G:U in seed) to the viral miRNAs using RNAhybrid (45).

### Statistical analysis

Statistical analysis was performed using GraphPad Prism v10.  Data are presented as average ± standard deviation (SD). For luciferase and qRT-PCR assays, differences between groups were analyzed using Student’s *t*-tests to determine statistical significance. For IFN response assays, dose responses were calculated as AUC, and two-tailed unpaired *t*-tests were used to determine significance. *P* values less than 0.05 were considered significant.

## SUPPLEMENTAL MATERIAL

Fig. S1 jvi.01579-25-s0001.eps Viral miRNA expression.

Fig. S2 jvi.01579-25-s0002.eps Construction of rLCV miRKO recombinant viruses.

Fig. S3 jvi.01579-25-s0003.eps rLCV gene expression in rBART miRKO rLCLs.

Fig. S4 jvi.01579-25-s0004.eps Luciferase reporter assays confirm viral miRNA targets.

Supplemental legends jvi.01579-25-s0005.docx Legends for Fig. S1 to S4.

Table S1 jvi.01579-25-s0006.docx Oligonucleotides used for cloning.

Table S2 jvi.01579-25-s0007.docx Predicted seed matches in 3′UTRs.

ASM does not own the copyrights to Supplemental Material that may be linked to, or accessed through, an article. The authors have granted ASM a non-exclusive, world-wide license to publish the Supplemental Material files. Please contact the corresponding author directly for reuse.
