# Supplementary material for: Inhibition of type I interferon signaling is a conserved function of gamma-herpesvirus-encoded microRNAs
Source: J Virol. 2025 Dec 31;100(2):e01579-25. doi: 10.1128/jvi.01579-25 (PMC12911870; doi:10.1128/jvi.01579-25)
Supplement: Supplemental legends — Legends for Fig. S1 to S4. [file jvi.01579-25-s0005.docx]

**Supplemental Figure 1. Viral miRNA expression.** A. and B. 293-ISRE or tRF-ISRE cells were transfected with indicated RRV miRNA expression vectors as in Figure 1B or 2A. Values are normalized to U6 or miR-16 and reported relative to miRNA levels in rhesus fibroblasts (RFs) infected with RRV for 72 hr. C. 293T cells were transfected with indicated rLCV miRNA expression vectors. Values are normalized to miR-16 and reported relative to viral miRNA levels in latently infected rLCLs. D. tRF cells were transfected with indicated rLCV miRNA expression vectors as in Figure 2E and 2F. Values are normalized to miR-16 and reported relative to viral miRNA levels in LCL8664. For A-D, total RNA was harvested 48 hr post-transfection and assessed for miRNA expression by qRT-PCR. Shown are the averages of at least four biological replicates with standard deviations.

**Supplemental Figure 2. Construction of rLCV miRKO recombinant viruses.** A. and B. Restriction enzyme digests of rLCV recombinants. BAC DNA was purified from independent bacterial clones and subject to restriction enzyme digest with BamHI. Banding patterns confirm intact BACs and are consistent with introduced mutations that disrupt rBHRF1 or rBART miRNA production. rLCV BHRF1 miRKO clone 1 and rLCV BART miRKO clone A86-1 were confirmed with Sanger sequencing and used for experiments. C. rLCV BHRF1 miRNA expression. The rLCV WT BHRF1 region as well as a synthesized rBHRF1 region with mutations within the rBHRF1 miRNAs were cloned into pcDNA3. Constructs were transfected into 293T cells and RNA harvested 48 hr. miRNA expression was evaluated by qRT-PCR. Values are normalized to miR-16 and reported relative to viral miRNA levels in rLCLs infected with the WT BAC strain. D. EBV miRNAs are not detectable in RM PBMCs. Two EBV-specific miRNAs (miR-BART8 and miR-BART9) were tested in RM PBMCs infected with P3HR1-derived rLCV recombinant viruses for 72 hr. Infections correspond to Figure 4. Values are normalized to miR-16 and reported relative to levels in EBV+ P3HR1 cells. E. rLCLs produced with P3HR1-derived WT rLCV do not harbor detectable EBV genomes. Genomic DNA was isolated from P3HR1 cells or rLCLs; EBV DNA was assessed using qPCR primers to LMP1. Values are normalized to GAPDH and reported relative to EBV DNA levels in P3HR1 cells.

**Supplemental Figure 3. rLCV gene expression in rBART miRKO rLCLs.** A-C. rEBER1, rBHRF1, and rBZLF1 expression levels in rLCLs established with WT or rBART miRKO viruses. Gene expression was evaluated by qRT-PCR and rLCLs tested in triplicate, corresponding to Figure 5. Values are normalized to GAPDH and reported relative to LCL8664.

**Supplemental Figure 4. Luciferase reporter assays confirm viral miRNA targets.** A-F. RM 3’UTRs for CHUK and IKBKB, human 3’UTRs for DAZAP2, SP100, and ZCCHC3, or the EBV BART19-indicator were tested in luciferase assays against indicated rLCV miRNA vectors. The corresponding EBV miRNA homolog that targets each human 3’UTR is listed. G. RRV miR-rR1-8 targets 3’UTRs with cognate miR-17/miR-373 seed match sites. 293T cells were co-transfected with 20 ng of firefly luciferase reporter (GL3 or the miR-17 indicator 17.5i), 20 ng renilla luciferase vector, and 250 ng of pcDNA3 or RRV8 vector. For all assays, 293T cells were co-transfected with 20 ng of luciferase reporter and 250 ng of indicated miRNA expression vector. Lysates were harvested 48-72 hr post-transfection and assayed for dual luciferase activity. Values are normalized to renilla luciferase as the internal control and are reported relative to pcDNA3 control vector. RLU = relative light units. *By Student’s t-test, p<0.05.
