# Supplementary material for: Inhibition of type I interferon signaling is a conserved function of gamma-herpesvirus-encoded microRNAs
Source: J Virol. 2025 Dec 31;100(2):e01579-25. doi: 10.1128/jvi.01579-25 (PMC12911870; doi:10.1128/jvi.01579-25)
Supplement: Table S1 — Oligonucleotides used for cloning. [file jvi.01579-25-s0006.docx]

**Table S1, Oligonucleotides used for cloning**

| **Viral miRNA** | **EBV/KSHV homolog** | **Seed mimic (cellular)** | **Cloning (Fwd Primer)** | **Cloning (Rev Primer)** |
| --- | --- | --- | --- | --- |
| rL1-5 | miR-BART3 | miR-29 family | published in Skalsky, 2014 |  |
| rL1-6 | miR-BART1 | miR-29 family | published in Skalsky, 2014 |  |
| rL1-8 | miR-BART5 | miR-18a | published in Skalsky, 2014 |  |
| rL1-16 | miR-BART20 | n/a | published in Skalsky, 2014 |  |
| rL1-9 | miR-BART17 | n/a | CACTCGAGCCCTCCTAACACCTA | CATCTAGAGTCATTCAACGTAGGG |
| rL1-13 | miR-BART7 | miR-136 or miR-378a | CACTCGAGGGTGCATGCATTTCTTATCGG | CATCTAGATGAGACTAGAGGCCCACAAA |
| rL1-15 | miR-BART12 | n/a | CACTCGAGGTAGCCGAGCATTGTTAGTT | CATCTAGAACCGGGTTCAAGATCATTTC |
| rL1-21 | None | n/a | CACTCGAGGGCTGAGCTGTGCAGTTT | CATCTAGAGGCTTCTGGAGAGCTGTGA |
| rL1-27 | miR-BART10 | n/a | CACTCGAGGTGCTGCTTTCTGGTTTCTA | CATCTAGATGAGAATCAACAGGCCAATC |
| rL1-29 | miR-BART19 | n/a | CACTCGAGTGTCCATTTCTTCGTGAGC | CATCTAGAGTCTGGACAGCAGCAAC |
| RRV1.9 | miR-K3 | n/a | CACTCGAGGGCACTCTCCTGTCTATTCT | CATCTAGATGTGCACATCTTTCAGTGACC |
| RRV2 | None | n/a | CACTCGAGATGCGCTGGATGATGTTTGG | CATCTAGATGAATCACCAAGAATACGGCCA |
| RRV3.12 | None | n/a | CACTCGAGCACCAAGCACCACATTTAC | CATCTAGACTAACGCAAAGGGCACTTAATTT |
| RRV4 | miR-K12 | n/a | CACTCGAGACGACTAAAGGTTTCTGTGGT | CATCTAGAGTCGCGTGCTAAGTGTTTTT |
| RRV8 | None | miR-17/373 family | CACTCGAGGACACCTCACGCACGCAA | CATCTAGACCTCCGTTCCGTTGTCTTCT |
| RRV13 | None | miR-363/367 family | CACTCGAGTATGGGCCGACTCGACTAAA | CAGCTAGCTGACCGTGTGCTGCTTAAC |
| RRV14 | None | n/a | CACTCGAGGTTAAGCAGCACACGGTCAC | CATCTAGAATCACCAAGTATCCCATCAACCT |
| RRV15 | miR-K10 | miR-142 | CACTCGAGGTCTCGTTACGATGTGCATTAAC | CAGCTAGCGTTCACCGCCAACAACAAA |
