# Supplementary material for: Inhibition of type I interferon signaling is a conserved function of gamma-herpesvirus-encoded microRNAs
Source: J Virol. 2025 Dec 31;100(2):e01579-25. doi: 10.1128/jvi.01579-25 (PMC12911870; doi:10.1128/jvi.01579-25)
Supplement: Table S2 — Predicted seed matches in 3′UTRs. [file jvi.01579-25-s0007.docx]

**Table S2, Predicted seed matches in 3’UTRs**

| **3'UTR** | **miRNA** | **Seed Match** | **Responds in Luciferase Assay?** |
| --- | --- | --- | --- |
| RM JAK1 | rrv-miR-rR1-8-3p | 7mer1A, 8mer1A | No |
| RM JAK1 | rlcv-miR-rL1-6-5p | 6mer | Yes |
| RM JAK1 | rlcv-miR-rL1-9-5p | 7mer | Yes |
| RM STAT1 | rlcv-miR-rL1-16-5p | 7mer | No |
| RM STAT1 | rlcv-miR-rL1-16-3p | 6mer, 6mer | No |
| RM STAT1 | rlcv-miR-rL1-9-5p | 7mer | No |
| RM STAT1 | rrv-miR-rR1-12-5p | 6mer | No |
| RM STAT1 | rrv-miR-rR1-13-3p | 6mer | No |
| RM IRF9 | rlcv-miR-rL1-9-5p | 7mer, 7mer1A, 7mer, 7mer | Yes |
| RM IRF9 | rlcv-miR-rL1-6-5p | 10mer1A | Yes |
| RM IRF9 | rlcv-miR-rL1-21-3p | 10mer1A | No |
| RM IRF9 | rlcv-miR-rL1-27-5p | 10mer1A | No |
| RM IRF9 | rlcv-miR-rL1-29-5p | 7mer | No |
| RM IRF9 | rrv-miR-rR1-9-5p | 7mer1A | No |
| RM IRF9 | rrv-miR-rR1-12-5p | 6mer | No |
| RM IRF9 | rrv-miR-rR1-8-3p | 7mer, 7mer | Yes |
| hIFNAR1 | rrv-miR-rR1-9-5p | 8mer1A* | No |
| hIFNAR1 | rrv-miR-rR1-3-3p | 8mer1A* | Yes |
| hIFNAR1 | rrv-miR-rR1-8-5p | 7mer* | No |
| hIFNAR1 | rrv-miR-rR1-8-3p | 7mer | No |
| hIFNAR1 | rlcv-miR-rL1-6-5p | 7mer1A* | No |
| hIFNAR1 | rlcv-miR-rL1-21-3p | 8mer* | No |
| hIFNAR1 | rlcv-miR-rL1-27-3p | 7mer1A | No |
| RM IFNAR2 | rrv-miR-rR1-3-3p | 6mer | No |
| RM IFNAR2 | rrv-miR-rR1-3-5p | 6mer | No |
| RM IFNAR2 | rlcv-miR-rL1-5-5p | 7mer1A, 8mer1A | No |
| RM IFNAR2 | rlcv-miR-rL1-5-3p | 6mer | No |
| RM IFNAR2 | rlcv-miR-rL1-6-5p | 7mer1A | No |
| RM IFNAR2 | rlcv-miR-rL1-6-3p | 6mer | No |
| RM IFNAR2 | rlcv-miR-rL1-8-5p | 6mer, 6mer, 7mer1A | Yes |
| RM IFNAR2 | rlcv-miR-rL1-15-5p | 8mer | No |
| RM IFNAR2 | rlcv-miR-rL1-29-5p | 6mer, 6mer, 6mer | No |
| RM IFNAR2 | rlcv-miR-rL1-29-3p | 9mer1A, 8mer1A | No |

*site occurs in RM IFNAR1 3’UTR
